# Elotuzumab, lenalidomide, and dexamethasone in RRMM: final overall survival results from the phase 3 randomized ELOQUENT-2 study

**DOI:** 10.1038/s41408-020-00357-4

**Published:** 2020-09-04

**Authors:** Meletios A. Dimopoulos, Sagar Lonial, Darrell White, Philippe Moreau, Katja Weisel, Jesus San-Miguel, Ofer Shpilberg, Sebastian Grosicki, Ivan Špička, Adam Walter-Croneck, Hila Magen, Maria-Victoria Mateos, Andrew Belch, Donna Reece, Meral Beksac, Andrew Spencer, Heather Oakervee, Robert Z. Orlowski, Masafumi Taniwaki, Christoph Röllig, Hermann Einsele, Morio Matsumoto, Ka Lung Wu, Kenneth C. Anderson, Ying-Ming Jou, Alex Ganetsky, Anil K. Singhal, Paul G. Richardson

**Affiliations:** 1 National and Kapodistrian University of Athens School of Medicine, Athens, Greece; 2 Winship Cancer Institute, Emory University, Atlanta, GA USA; 3 Queen Elizabeth II Health Sciences Centre and Dalhousie University, Halifax, NS Canada; 4 University Hospital, Nantes, France; 5 University Medical Center of Hamburg-Eppendorf, Hamburg, Germany; 6 Clínica Universidad de Navarra, Centro de Investigación Médica Aplicada, IDISNA, CIBERONC, Pamplona, Spain; 7 Institute of Haematology, Assuta Medical Centers, Tel Aviv, Israel; 8 Medical University of Silesia, Katowice, Poland; 9 Charles University in Prague and General Teaching Hospital, Prague, Czech Republic; 10 Medical University of Lublin, Lublin, Poland; 11 Department of Hematology Chaim Sheba Medical Center, Ramat-Gan, Sackler Faculty of Medicine, Tel Aviv University, Tel Aviv, Israel; 12 University Hospital of Salamanca–Instituto de Investigación Biomédica de Salamanca, Centro de Investigación del Cáncer-IBMCC (USAL-CSIC), Salamanca, Spain; 13 Cross Cancer Institute and University of Alberta, Edmonton, AB Canada; 14 Princess Margaret Hospital, Toronto, ON Canada; 15 Ankara University, Ankara, Turkey; 16 Alfred Health–Monash University, Melbourne, VIC Australia; 17 Barts Health NHS Trust, London, UK; 18 The University of Texas MD Anderson Cancer Center, Houston, TX USA; 19 Kyoto Prefectural University of Medicine, Kyoto, Japan; 20 Universitätsklinikum der Technischen Universität, Dresden, Germany; 21 Universitätsklinikum Würzburg, Würzburg, Germany; 22 National Hospital Organization Shibukawa Medical Center, Shibukawa, Japan; 23 Ziekenhuis Netwerk Antwerpen Stuivenberg, Antwerp, Belgium; 24 Dana–Farber Cancer Institute, Boston, MA USA; 25 Bristol-Myers Squibb Company, Princeton, NJ USA; 26 AbbVie Biotherapeutics, Redwood City, CA USA

**Keywords:** Cancer therapy, Myeloma

## Abstract

Prolonging overall survival (OS) remains an unmet need in relapsed or refractory multiple myeloma (RRMM). In ELOQUENT-2 (NCT01239797), elotuzumab plus lenalidomide/dexamethasone (ERd) significantly improved progression-free survival (PFS) versus lenalidomide/dexamethasone (Rd) in patients with RRMM and 1–3 prior lines of therapy (LoTs). We report results from the pre-planned final OS analysis after a minimum follow-up of 70.6 months, the longest reported for an antibody-based triplet in RRMM. Overall, 646 patients with RRMM and 1–3 prior LoTs were randomized 1:1 to ERd or Rd. PFS and overall response rate were co-primary endpoints. OS was a key secondary endpoint, with the final analysis planned after 427 deaths. ERd demonstrated a statistically significant 8.7-month improvement in OS versus Rd (median, 48.3 vs 39.6 months; hazard ratio, 0.82 [95.4% Cl, 0.68–1.00]; *P* = 0.0408 [less than allotted *α* of 0.046]), which was consistently observed across key predefined subgroups. No additional safety signals with ERd at extended follow-up were reported. ERd is the first antibody-based triplet regimen shown to significantly prolong OS in patients with RRMM and 1–3 prior LoTs. The magnitude of OS benefit was greatest among patients with adverse prognostic factors, including older age, ISS stage III, IMWG high-risk disease, and 2–3 prior LoTs.

## Introduction

Despite the introduction of novel and highly effective front-line therapies, nearly all patients with multiple myeloma (MM) eventually relapse and become refractory to treatment. As such, the 5-year overall survival (OS) rate remains low, at ~50%^1^. Outcomes are particularly poor for patients with relapsed or refractory (RR) MM, as durability of response decreases with successive lines of therapy^2–6^. Therefore, identifying therapeutic options that prolong OS in RRMM remains critical. Treatment regimens that incorporate monoclonal antibodies have demonstrated durable responses and extended progression-free survival (PFS); however, final OS analyses for these therapies have not yet been reported^7–9^.

Elotuzumab is a first-in-class humanized immunoglobulin G1 immunostimulatory monoclonal antibody that targets signaling lymphocytic activation molecule family member 7 (SLAMF7)^10^. SLAMF7 is a cellular glycoprotein that is highly expressed on MM cells, natural killer (NK) cells, and some other immune cells, but has minimal expression on normal tissues^10^. Elotuzumab has multiple mechanisms of action against MM cells, including direct NK cell activation, NK cell–mediated antibody-dependent cellular cytotoxicity and macrophage-mediated antibody-dependent cellular phagocytosis^10–13^. Lenalidomide, an immunomodulatory drug, synergizes with elotuzumab by increasing cytokine production (e.g., interleukin-2 and tumor necrosis factor α) and enhancing the elotuzumab-mediated NK cell killing of MM cells^14^. The phase 3 ELOQUENT‑2 study assessed the efficacy and safety of elotuzumab plus lenalidomide and dexamethasone (ERd) versus lenalidomide and dexamethasone (Rd) alone in patients with RRMM and 1–3 prior lines of therapy^15^. At the primary analysis of ELOQUENT-2, patients treated with ERd had a 30% reduction in the risk of progression or death versus Rd (median PFS, 19.4 months vs 14.9 months; hazard ratio [HR], 0.70; 95% confidence interval [CI], 0.57–0.85; *P* < 0.001)^15^. In addition, ERd was associated with an improved overall response rate (ORR) versus Rd (ERd, 79%; Rd, 66%; odds ratio: 1.9; 95% CI, 1.4–2.8; *P* < 0.001)^15^. Based on these results, ERd received approval for the treatment of patients with RRMM and 1–3 prior therapies in the United States and ≥1 prior therapy in the European Union. At the 3-, 4-, and 5-year follow-up of ELOQUENT-2, the PFS benefit with ERd was sustained and durable, and no new relevant safety information was identified^7,16,17^. A pre-planned interim analysis of OS after a minimum follow-up of 3 years showed a trend in favor of ERd versus Rd (median OS: ERd, 43.7 months; Rd, 39.6 months; HR, 0.77; 95% CI, 0.61–0.97), although OS curves at that time were not fully mature, limiting full interpretation^16^. Here, we present the final OS analysis of ELOQUENT-2 after the longest follow-up to date for any antibody-based triplet in patients with RRMM and 1–3 prior lines of therapy (minimum of 70.6 months).

## Methods

### Study design and patients

ELOQUENT-2 (registered at www.clinicaltrials.gov as NCT01239797) is a phase 3, open-label, multicenter, randomized study that evaluated ERd versus Rd in patients with RRMM. The study design has been previously described^15^. Eligible patients were ≥18 years of age and had MM, measurable disease, and 1–3 prior lines of therapy with documented progression after their most recent therapy. All patients had Eastern Cooperative Oncology Group performance status ≤2 and creatinine clearance ≥30 mL/min. Per protocol, no more than 10% of patients with prior lenalidomide were permitted to be enrolled, which was to increase the likelihood that subjects at study enrollment were lenalidomide-sensitive. Patients were randomized via an interactive voice response system in a 1:1 ratio to receive ERd or Rd in 28-day cycles until disease progression, unacceptable toxicity, or discontinuation. Randomization was stratified by β2 microglobulin levels (<3.5 vs ≥3.5 mg/L), prior lines of therapy (1 vs 2–3), and prior use of immunomodulatory drugs (none vs thalidomide only vs other). For analysis of OS according to disease risk, the International Myeloma Working Group (IMWG) consensus recommendations on risk stratification were used; these include high risk (International Staging System [ISS] stage II or III and *t*[4;14] or del(17p) abnormality), standard risk (patients not meeting the definition of high or low risk) or low risk (ISS stage I or II, absence of *t*[4;14], del(17p) and 1q21 abnormalities, and age <55 years)^18^.

The study was approved by the Institutional Review Board/Independent Ethics Committee at each site before initiation. The study was conducted in accordance with Good Clinical Practice, as defined by the International Conference on Harmonization, and the Declaration of Helsinki. All patients provided written informed consent. All authors had access to the clinical trial data.

### Study endpoints and assessments

The co-primary endpoints were PFS (time from randomization to first documented tumor progression or death) and ORR (partial response or better), which have been previously described^15^. OS was a key secondary endpoint, defined as time from randomization to the date of death from any cause (Supplementary Appendix). Exploratory endpoints included safety, time to tumor response, and duration of response (DoR). Adverse events (AEs) were coded using the Medical Dictionary for Regulatory Activities (MedDRA), version 21.1, and graded by the National Cancer Institute Common Terminology Criteria for Adverse Events (CTCAE), version 3.0. Cytogenetic assessments by karyotyping and fluorescence in situ hybridization was performed by a central laboratory on samples collected at screening. A positive score for each tested abnormality was assigned based on identifying at least one abnormal cell out of a minimum of 200 cells examined.

### Statistics

The final OS analysis was planned after a pre-specified 427 deaths had occurred. OS was tested hierarchically to the co-primary endpoints of PFS and ORR to preserve the experiment-wise type I error at the 5% level. The overall significance level (*α*) at which OS was tested depended on, and corresponded to, the significance level of the individual co-primary endpoints (PFS, 0.045; ORR, 0.005). Since the interim PFS and ORR results were both statistically significant^15^, their combined total *α* of 0.05 was passed down to OS, followed by group sequential tests for the multiple OS analyses. The two-sided *α* of 0.046 for the final analysis of OS was determined using an O’Brien–Fleming spending function, based on the 295 deaths observed at interim analysis in the group sequential tests (two-sided *α* of 0.014)^16^. The Kaplan–Meier method was used to estimate the distribution of OS; HRs for ERd versus Rd were estimated with a stratified Cox proportional hazards model. Comparisons between treatment arms were based on the stratified log-rank test; stratification factors were the same as those used in randomization. PFS analyses at the time of the final OS analysis were exploratory.

### Data sharing statement

Bristol–Myers Squibb Company’s policy on data sharing may be found at https://www.bms.com/researchers-and-partners/independent-research/data-sharing-request-process.html.

## Results

### Baseline patient characteristics

In total, 646 patients were randomized (ERd, *n* = 321; Rd, *n* = 325) and 635 received treatment with ERd (*n* = 318) or Rd (*n* = 317). The baseline demographics and disease characteristics have been previously described^15^ and were generally balanced between treatment arms, including the proportion of patients with prior lenalidomide (Supplementary Table S1). The median (range) age of patients was 66 (37–91) years and 20% (*n* = 129) were ≥75 years of age. Similar proportions of patients had ISS stage III disease (ERd, 21% [*n* = 66]; Rd, 21% [*n* = 68]) at diagnosis, and 35% of patients in each arm (ERd, *n* = 113; Rd, *n* = 114) were refractory to their most recent line of therapy. Median time from disease diagnosis to randomization was 3.5 years across both treatment arms.

### Patient disposition and drug exposure

**Fig. 1:**
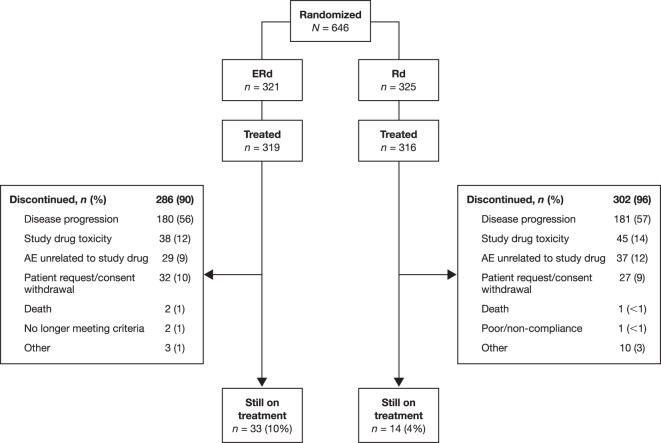
Patient disposition. One patient randomized to treatment with ERd received Rd. *AE* adverse event, *ERd* elotuzumab plus lenalidomide and dexamethasone, *Rd* lenalidomide and dexamethasone.

After a minimum follow-up of 70.6 months (i.e., time from last patient randomized [November 16, 2012] to clinical data cut-off [October 3, 2018]), more than twice as many patients remained on treatment with ERd (*n* = 33 [10%]) versus Rd (*n* = 14 [4%]; Fig. 1). The most common reasons for discontinuation were disease progression (ERd, 56%; Rd, 57%) and study drug toxicity (ERd, 12%; Rd, 14%). The median drug exposure was 5 months longer in the ERd arm (17 months vs 12 months), with a median (range) of 19 (1–91) and 14 (1–83) treatment cycles in the ERd and Rd arms, respectively.

### Efficacy

#### Overall survival

**Fig. 2:**
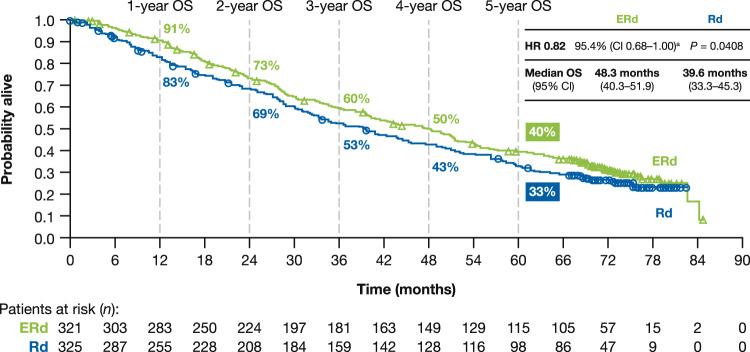
Overall survival. ^a^Upper limit of the 95.4% CI is <1 when calculated to 3 decimal places: HR, 0.820; 95.4% CI, 0.676–0.995; *P* = 0.0408. *CI* confidence interval, *ERd* elotuzumab plus lenalidomide and dexamethasone, *HR* hazard ratio, *OS* overall survival, *Rd* lenalidomide and dexamethasone.

A total of 437 deaths (212 [66%] deaths in the ERd arm and 225 [69%] deaths in the Rd arm) had occurred at the time of the final OS analysis. Median OS was 8.7 months longer with the addition of elotuzumab to Rd, at 48.3 months with ERd and 39.6 months with Rd. Early and sustained separation of OS curves was seen over time (1-year: ERd, 91%; Rd, 83%; 2-year: ERd, 73%; Rd, 69%; 3-year: ERd, 60%; Rd, 53%; 4-year: ERd, 50%; Rd, 43%; 5-year: ERd, 40%; Rd, 33%), with an 18% reduction in the risk of death with ERd versus Rd (HR, 0.82; 95.4% Cl, 0.68–1.00; *P* = 0.0408, less than the allotted *α*; Fig. 2).

#### Subgroup analyses of overall survival

**Fig. 3:**
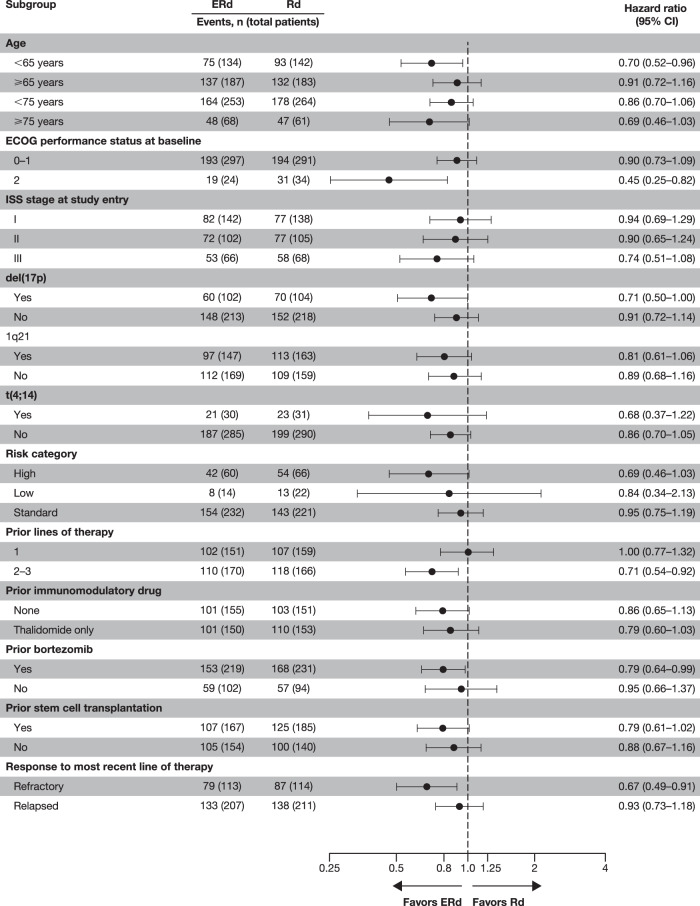
Overall survival by predefined subgroups. *CI* confidence interval, *ERd* elotuzumab plus lenalidomide and dexamethasone, *ISS* International Staging System, *Rd* lenalidomide and dexamethasone.

The OS benefit observed with ERd was maintained across most clinically relevant subgroups of interest (Fig. 3). Median OS with ERd versus Rd was 17.4 months longer with ERd in patients with 2–3 prior lines of therapy (51.0 vs 33.6 months; HR, 0.71; 95% CI, 0.54–0.92) and similar in patients with 1 prior line of therapy (43.7 vs 44.1 months; HR, 1.00; 95% CI, 0.77–1.32). When analyzed by age, median OS was 21.1 months longer with ERd versus Rd in patients ≥75 years (48.5 vs 27.4 months; HR, 0.69; 95% CI, 0.46–1.03), 4.2 months longer in patients <75 years of age (47.9 vs 43.7 months; HR, 0.86; 95% CI, 0.70–1.06), and 15.8 months longer in patients <65 years (63.5 vs 47.7 months; HR, 0.70; 95% CI, 0.52–0.96). For patients refractory to their most recent therapy, median OS with ERd was 14.5 months longer than with Rd (40.4 vs 25.9 months; HR, 0.67; 95% CI, 0.49–0.91); for patients with relapsed disease after their last therapy, median OS with ERd was 3.5 months longer than with Rd (51.2 vs versus 47.7 months; HR, 0.93; 95% CI, 0.73–1.18). In addition, ERd was associated with an OS benefit in other clinically relevant subgroups of interest, including patients with high-risk disease (median, 29.8 vs 24.8 months; HR, 0.69; 95% CI, 0.46–1.03), del(17p) mutations in ≥1 cell (median, 50.1 vs 36.4 months; HR, 0.71; 95% CI, 0.50–1.00), prior stem cell transplantation (median, 49.6 vs 41.3 months; HR, 0.79; 95% CI, 0.61–1.02), or ISS stage III disease (median, 21.7 vs 14.0 months; HR, 0.74; 95% CI, 0.51–1.08).

#### Subsequent therapy

After discontinuation of study therapy, 60% (192/321) of ERd-treated patients and 66% (213/325) of Rd-treated patients received subsequent systemic therapy (Table 1). The most common subsequent therapies were similar in both treatment arms, and included bortezomib (ERd, 38%; Rd, 42%), cyclophosphamide (ERd, 30%; Rd, 30%), and pomalidomide (ERd, 26%; Rd, 29%). The most common reason for subsequent systemic therapy in both treatment arms was documented disease progression (ERd, 53%; Rd, 55%).

**Table 1:** Subsequent therapy.

	ERd (*n* = 321)	Rd (*n* = 325)
Any subsequent therapy	192 (60)	213 (66)
Most common subsequent therapy^**a**^		
Dexamethasone	170 (53)	178 (55)
Bortezomib	122 (38)	137 (42)
Cyclophosphamide	96 (30)	99 (30)
Pomalidomide	82 (26)	94 (29)
Lenalidomide	57 (18)	71 (22)
Carfilzomib	47 (15)	45 (14)
Melphalan	32 (10)	35 (11)
Bendamustine	30 (9)	27 (8)
Thalidomide	30 (9)	42 (13)
Daratumumab	28 (9)	38 (12)
Doxorubicin	19 (6)	29 (9)
Etoposide	16 (5)	17 (5)
Prednisolone	16 (5)	14 (4)
Prednisone	15 (5)	25 (8)
Cisplatin	15 (5)	15 (5)

Data are *n* (%).

*ERd* elotuzumab plus lenalidomide and dexamethasone, *Rd* lenalidomide and dexamethasone.

^a^≥5% of patients in the ERd arm.

#### Updated PFS

**Fig. 4:**
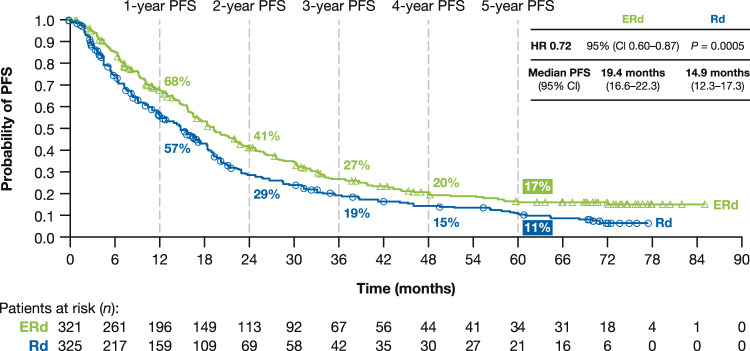
Progression-free survival. *CI* confidence interval, *ERd* elotuzumab plus lenalidomide and dexamethasone, *HR* hazard ratio, *PFS* progression-free survival, *Rd* lenalidomide and dexamethasone.

At clinical data cut-off (minimum follow-up: 70.6 months), per independent review committee (IRC) there were 233 (73%) PFS events in the ERd arm and 245 (75%) in the Rd arm (Fig. 4). The PFS benefit with ERd versus Rd was observed early and sustained through extended follow-up (1-year: ERd, 68%; Rd, 57%; 2-year: ERd, 41%; Rd, 29%; 3-year: ERd, 27%; Rd, 19%; 4-year: ERd, 20%; Rd, 15%; 5-year: ERd, 17%; Rd, 11%). Median PFS had been reached at the time of the primary analysis^15^. A similar benefit was seen with the intent-to-treat definition of PFS, as well as per investigator.

#### Duration of response

The median DoR per IRC was 21.9 months (95% CI, 18.4–26.6) for ERd and 17.1 months (95% CI, 15.2–19.4) for Rd (Supplementary Fig. 1).

### Safety

The safety profile of ERd remained consistent with the primary analysis, with no additional safety signals identified with extended follow-up. Among all treated patients, fewer deaths occurred in the ERd (67%) arm versus the Rd (71%) arm. The main cause of death was disease progression in both treatment arms (ERd, 41%; Rd, 45%; Supplementary Table 2). Safety data after extended follow-up were similar between treatment arms (Table 2). The most common all-cause AEs of any grade were diarrhea (ERd, 50%; Rd, 39%), fatigue (ERd, 49%; Rd, 41%), anemia (ERd, 44%; Rd, 38%), pyrexia (ERd, 41%; Rd, 26%), constipation (ERd, 36%; Rd, 28%), and neutropenia (ERd, 36%; Rd, 43%). All-cause grade 3–4 AEs occurred in 77% (ERd) and 68% (Rd) of patients. Hematological AEs (blood and lymphatic system organ class) were reported in 67% of patients in the ERd arm and 63% of patients in the Rd arm. The incidence of infections of any grade was 84% (ERd) and 75% (Rd), with pneumonia reported in 22% and 16% of patients, respectively. Infusion reactions with ERd were experienced by 11% (*n* = 35) of patients; all were grade 1–2 (*n* = 30, 9%) or grade 3 (*n* = 5, 2%). All-cause AEs leading to discontinuation occurred in 36% (ERd, *n* = 114) and 33% (Rd, *n* = 104) of patients, most commonly infections (ERd, 6% [*n* = 18]; Rd, 5% [*n* = 17]). Grade 3–4 AEs leading to discontinuation occurred in 21% (ERd, *n* = 66) and 20% (Rd, *n* = 63) of patients. Serious AEs were reported in 75% and 61% of patients in the ERd arm and Rd arm, respectively. The most common serious AE in each arm was pneumonia, occurring in 17% (ERd, *n* = 54) and 13% (Rd, *n* = 40) of patients. The proportion of patients who experienced a second primary malignancy (SPM) was 12% in the ERd arm and 9% in the Rd arm. After adjustment for drug exposure, the incidence rate of SPMs was 5.1/100 patient-years in the ERd arm and 4.0/100 patient-years in the Rd arm. The most common SPMs were basal cell carcinoma and squamous cell carcinoma of the skin (for each: ERd, 3%; Rd, 2%).

**Table 2:** All-cause adverse events.

	ERd (*n* = 318)			Rd (*n* = 317)		
	Any grade	Grade 3–4	Events/100 PY^a^	Any grade	Grade 3–4	Events/100 PY^b^
Event	316 (99)	244 (77)	NR	314 (99)	217 (68)	NR
AEs in ≥30% of patients						
Diarrhea	160 (50)	24 (8)	NR	125 (39)	17 (5)	NR
Fatigue	155 (49)	32 (10)	NR	131 (41)	27 (9)	NR
Anemia	139 (44)	57 (18)	NR	120 (38)	53 (17)	NR
Pyrexia	129 (41)	11 (3)	NR	81 (26)	11 (3)	NR
Constipation	115 (36)	4 (1)	NR	89 (28)	1 (<1)	NR
Neutropenia	114 (36)	86 (27)	NR	137 (43)	109 (34)	NR
Cough	109 (34)	1 (<1)	NR	62 (20)	0	NR
Back pain	108 (34)	19 (6)	NR	98 (31)	17 (5)	NR
Muscle spasm	100 (31)	2 (1)	NR	85 (27)	3 (1)	NR
Peripheral edema	95 (30)	4 (1)	NR	78 (25)	1 (<1)	NR
AEs/AE categories of special interest						
Infections	267 (84)	112 (35)	199	239 (75)	85 (27)	185
Pneumonia	69 (22)	48 (15)	13	51 (16)	33 (10)	12
Herpes zoster	23 (7)	6 (2)	3	7 (2)	2 (1)	1
Sepsis^c^	13 (4)	11 (3)	NR	13 (4)	6 (2)	NR
Renal and urinary disorders	87 (27)	17 (5)	17	60 (19)	15 (5)	17
Cardiac disorders	75 (24)	18 (6)	17	59 (19)	24 (8)	18
Lymphopenia	41 (13)	27 (8)	12	23 (7)	12 (4)	11
SPMs	39 (12)	NR	5^d^	28 (9)	NR	4^e^
Basal cell carcinoma	11 (3)	NR	1^d^	6 (2)	NR	1^e^
Squamous cell carcinoma of the skin	10 (3)	NR	1^d^	6 (2)	NR	1^e^
Total serious AEs	238 (75)	169 (53)	NR	194 (61)	127 (40)	NR

Data are *n* (%).

*AE* adverse event, *ERd* elotuzumab plus lenalidomide and dexamethasone, *NR* not reported, *PY* patient-years, *Rd* lenalidomide and dexamethasone, *SPM* second primary malignancy.

^a^746.70 PY.

^b^543.77 PY.

^c^Includes urosepsis, bacterial, biliary, neutropenic, pneumococcal, pseudomonal, pulmonary, device-related, and staphylococcal sepsis.

^d^Adjusted based on 1166.98 PY of follow-up.

^e^Adjusted based on 1050.78 PY of follow-up.

## Discussion

The final OS analysis of the ELOQUENT-2 study demonstrates that the addition of elotuzumab to Rd results in a significant improvement in OS in patients with MM who received 1–3 prior lines of therapy. Patients treated with ERd had an 8.7-month increase in median OS compared with those receiving Rd (48.3 months vs 39.6 months). This represented a statistically significant reduction in the risk of death for ERd over Rd, with an HR of 0.82 (95.4% Cl, 0.676–0.995 when given to three decimal places; *P* = 0.0408, less than the allotted α). Furthermore, ERd demonstrated an acceptable safety profile, with no new safety signals detected.

The OS benefits observed with ERd were maintained across multiple predefined subgroups generally associated with poorer outcomes, including among patients with multiple prior lines of therapy, older patients, patients with ISS stage III disease, and those refractory to their most recent therapy. ERd was associated with a 17.4-month increase in median OS versus Rd in patients with 2–3 prior lines of therapy, a notable finding as MM typically becomes more difficult to treat with subsequent relapses. In the Rd arm, patients who received one prior therapy had a longer median OS (44.1 months) than those who received 2–3 prior therapies (33.6 months) and the overall population (39.6 months). In the ERd arm, patients with one prior therapy had a shorter median OS (43.7 months) than those who received 2–3 prior lines (51.0 months) and the overall population (48.3 months). As a result, the ERd/Rd hazard ratio was observed to be 1.00 among patients with one prior therapy, which was much greater than that of the overall population (0.82). This finding may be related to numerical imbalances in baseline characteristics within the one prior therapy subgroup that appear to delineate an unfavorable disease profile in patients treated with ERd: a numerically higher percentage of patients treated with ERd versus Rd were ≥75 years old, had renal impairment, refractory disease, and/or no prior stem cell transplant. In addition, a lower percentage of patients treated with ERd received subsequent therapy. Another notable finding was the 21.1 month increase in median OS in elderly patients (≥75 years) who received ERd compared with Rd. Elderly patients are more susceptible to treatment-related toxicities and are less likely to tolerate such toxicities due to frailty and/or comorbidities^19^. As such, and owing to difficulty in devising treatment regimens that optimize efficacy without a significant toxicity detriment, elderly patients often have poor outcomes. It should, however, be noted that given the relatively small number of elderly patients in this study (≥75 years, *n* = 129; <75 years, *n* = 517), this finding should be interpreted with caution. When analyzed by age group, median OS in the ERd arm was similar between patients aged <75 (47.9 months) and ≥75 (48.5 months) years, but was different in the Rd arm: 43.7 and 27.4 months for patients aged <75 versus ≥75 years, respectively. As a result, the ERd/Rd hazard ratio appeared smaller in the older age group (0.69) than in the overall population (0.82). The lower median OS in the older patients within the Rd arm may be related to the numerically higher percentage of patients in this group who had certain adverse baseline disease characteristics such as more lines of prior therapy, ISS stage II or III disease, lytic bone lesions, and/or plasmacytoma.

ERd was associated with additional OS benefits in patients with IMWG high-risk disease (median, 29.8 vs 24.8 months), disease refractory to last prior therapy (median, 40.4 vs 25.9 months), ISS stage III disease (median, 21.7 vs 14.0 months) and adverse cytogenetic abnormalities, further highlighting the clinical benefit of ERd for patients with RRMM and adverse prognostic factors. Prior therapies and baseline characteristics were similar between the ERd and Rd arms, facilitating comparison and interpretation of the OS benefit with ERd over Rd. Further, the median OS with Rd in ELOQUENT-2 was similar to that reported in other phase 3 studies in RRMM^20–22^, suggesting that the OS benefit with ERd treatment was not due to poor outcomes in the Rd arm. In light of these results, ERd therapy may particularly benefit patients with characteristics associated with poorer outcomes, particularly elderly patients who may have received multiple prior lines of therapy, as well as patients with ISS stage III disease, and patients refractory to their most recent therapy.

ELOQUENT-2 is the first study to demonstrate a significant OS advantage with an antibody-based triplet regimen in patients with RRMM. Further, these data represent the first mature OS data from ELOQUENT-2, building on previous interim results to better define the OS benefit of adding elotuzumab to Rd in different patient subgroups^16^. The data reported here complement preliminary OS findings from the phase 3 ELOQUENT-3 study (NCT02654132) of elotuzumab plus pomalidomide and dexamethasone in patients with relapsed and refractory MM and ≥2 prior lines of therapy (including lenalidomide and a proteasome inhibitor)^8^. Notably, no imbalances in subsequent therapies were observed between treatment arms in ELOQUENT-2, strengthening the conclusion that the OS benefit observed in ELOQUENT-2 was a direct result of the study therapies, and that elotuzumab-induced immunostimulation may specifically contribute to long-term disease control in RRMM.

Final OS data with the proteasome inhibitor carfilzomib have been reported in two phase 3 studies in patients with RRMM and 1–3 prior lines of therapy. The ENDEAVOR study (NCT01568866) reported a 7.6-month increase in median OS with carfilzomib plus dexamethasone versus bortezomib plus dexamethasone (Vd)^21^. In the ASPIRE study (NCT01080391), carfilzomib plus Rd (KRd) was associated with a 7.9-month increase in median OS versus Rd alone^20^. The magnitude of OS benefit observed with ERd is therefore comparable to the findings reported in both the ASPIRE and ENDEAVOR studies. Interestingly, data from the ASPIRE study show a greater benefit in median OS with KRd treatment in first relapse (11.4-month benefit), than in later relapse, demonstrating the potential benefits of using novel regimens early in the course of treatment. In contrast, the OS benefit with ERd over Rd in the present study was greater among patients with 2–3 prior lines of therapy (17.4-month benefit) than in first relapse. This finding with ERd is notable given the remaining unmet need for highly effective regimens during later relapse, where the ability to achieve disease control may diminish with each subsequent therapy. OS results from other phase 3 studies in RRMM, such as CASTOR (daratumumab plus Vd vs Vd; NCT02136134)^23^ and POLLUX (daratumumab plus Rd [DRd] vs Rd; NCT02076009)^9^ have yet to be reported, but will provide further insight into the clinical benefit of novel triplet therapies upon maturation of the data.

The myeloma treatment landscape has continued to evolve since the conception and conduct of this trial, and few patients in ELOQUENT-2 had received previous treatment with lenalidomide. Therefore, a limitation of this trial is that prior treatments received by patients in this trial may no longer reflect real-world clinical practice. However, given the length of follow-up required for assessing the significance of OS benefits, this limitation may apply to many clinical trials in RRMM.

Prolonged treatment with ERd was well tolerated and no new safety signals were identified. The higher rates of special interest AEs in the ERd arm may be a reflection of the longer duration of treatment in this arm, as shown by the similar exposure-adjusted rates. Notably, the exposure-adjusted rates of SPMs were similar between treatment arms. There was a somewhat higher incidence of infections and pneumonia in the ERd arm; however, we do not recommend routine prophylactic measures as no evidence exists suggesting that prophylaxis against infections in patients receiving ERd would aid in reducing the frequency or severity of infections. The addition of elotuzumab to Rd did not appear to result in a notable incremental increase in the incidence of any particular grade 3–4 AEs, and overall, despite having a longer duration of follow-up, the incidence of grade 3–4 AEs in ELOQUENT-2 remained similar to other studies investigating Rd-based triplet regimens in RRMM (i.e., ASPIRE^20^ and POLLUX^9^). The addition of elotuzumab to Rd appeared to have tolerable effects on hematological toxicity as neutropenia occurred in fewer patients treated with ERd (36%) versus Rd (43%). In contrast, the incidence of neutropenia was higher in the experimental arms of ASPIRE (KRd, 40%; Rd, 35%)^20^ and POLLUX (DRd, 61%; Rd, 45%)^9^. This low incidence of hematological toxicity supports the use of ERd in elderly or frail patients.

## Conclusions

Treatment with ERd demonstrated a statistically significant and clinically meaningful 18% reduction in the risk of death versus Rd in patients with MM who received 1–3 prior therapies, with an 8.7-month increase in median OS versus Rd. ERd appears most notably to improve OS versus Rd in patients with adverse disease features, including those with later relapse, high-risk disease, advanced disease stage, and older age. The durable and sustained efficacy of ERd, combined with an acceptable long-term safety and tolerability profile, supports this regimen as a standard for care for patients with RRMM and 1–3 prior lines of therapy.

## Supplementary information

Supplementary Appendix

Supplemental Figure 1

## References

[1] Howlader, N. et al. *SEER Cancer Statistics Review, 1975–2016*. Bethesda, MD; 2019.

[2] Kumar SK (2012). Risk of progression and survival in multiple myeloma relapsing after therapy with IMiDs and bortezomib: a multicenter international myeloma working group study. Leukemia.

[3] Kumar SK (2017). Natural history of relapsed myeloma, refractory to immunomodulatory drugs and proteasome inhibitors: a multicenter IMWG study. Leukemia.

[4] Usmani S (2016). Analysis of real-world data on overall survival in multiple myeloma patients with ≥3 prior lines of therapy including a proteasome inhibitor (PI) and an immunomodulatory drug (IMiD), or double refractory to a PI and an IMiD. Oncologist.

[5] Yong K (2016). Multiple myeloma: patient outcomes in real-world practice. Br. J. Haematol..

[6] Kumar SK (2004). Clinical course of patients with relapsed multiple myeloma. Mayo Clin. Proc..

[7] Lonial, S. et al. Extended 5-y follow-up (FU) of phase 3 ELOQUENT-2 study of elotuzumab + lenalidomide/dexamethasone (ELd) vs Ld in relapsed/refractory multiple myeloma (RRMM). In Proc. American Society of Clinical Oncology (ASCO) Annual Meeting; 1–5 June 2018; Chicago, IL. 8040.

[8] Dimopoulos, M. A. et al. Elotuzumab plus pomalidomide and dexamethasone for relapsed/refractory multiple myeloma: efficacy results after additional follow-up of the phase 2, randomized ELOQUENT-3 study. European Hematology Association (EHA) Annual Meeting; 13–16 June 2019; Amsterdam, The Netherlands. PS1370.

[9] Dimopoulos MA (2018). Daratumumab plus lenalidomide and dexamethasone versus lenalidomide and dexamethasone in relapsed or refractory multiple myeloma: updated analysis of POLLUX. Haematologica.

[10] Hsi ED (2008). CS1, a potential new therapeutic antibody target for the treatment of multiple myeloma. Clin. Cancer Res..

[11] Tai YT (2008). Anti-CS1 humanized monoclonal antibody HuLuc63 inhibits myeloma cell adhesion and induces antibody-dependent cellular cytotoxicity in the bone marrow milieu. Blood.

[12] Collins SM (2013). Elotuzumab directly enhances NK cell cytotoxicity against myeloma via CS1 ligation: evidence for augmented NK cell function complementing ADCC. Cancer Immunol. Immunother..

[13] Kurdi AT (2018). Antibody-dependent cellular phagocytosis by macrophages is a novel mechanism of action of elotuzumab. Mol. Cancer Ther..

[14] Balasa B (2015). Elotuzumab enhances natural killer cell activation and myeloma cell killing through interleukin-2 and TNF-alpha pathways. Cancer Immunol. Immunother..

[15] Lonial S (2015). Elotuzumab therapy for relapsed or refractory multiple myeloma. N. Engl. J. Med..

[16] Dimopoulos MA (2017). Elotuzumab plus lenalidomide/dexamethasone for relapsed or refractory multiple myeloma: ELOQUENT-2 follow-up and post-hoc analyses on progression-free survival and tumour growth. Br. J. Haematol..

[17] Dimopoulos MA (2018). Elotuzumab plus lenalidomide and dexamethasone in relapsed/refractory multiple myeloma: extended 4-year follow-up and analysis of relative progression-free survival from the randomized ELOQUENT-2 trial. Cancer.

[18] Chng WJ (2014). IMWG consensus on risk stratification in multiple myeloma. Leukemia.

[19] Rosko A, Giralt S, Mateos MV, Dispenzieri A (2017). Myeloma in elderly patients: when less is more and more is more. Am. Soc. Clin. Oncol.Educ. Book.

[20] Siegel DS (2018). Improvement in overall survival with carfilzomib, lenalidomide, and dexamethasone in patients with relapsed or refractory multiple myeloma. J. Clin. Oncol..

[21] Dimopoulos MA (2017). Carfilzomib or bortezomib in relapsed or refractory multiple myeloma (ENDEAVOR): an interim overall survival analysis of an open-label, randomised, phase 3 trial. Lancet Oncol..

[22] Dimopoulos MA (2009). Long-term follow-up on overall survival from the MM-009 and MM-010 phase III trials of lenalidomide plus dexamethasone in patients with relapsed or refractory multiple myeloma. Leukemia.

[23] Moreau P (2016). Oral ixazomib, lenalidomide, and dexamethasone for multiple myeloma. N. Engl. J. Med..

