# Supplementary material for: Elotuzumab, lenalidomide, and dexamethasone in RRMM: final overall survival results from the phase 3 randomized ELOQUENT-2 study
Source: Blood Cancer J. 2020 Sep 4;10(9):91. doi: 10.1038/s41408-020-00357-4 (PMC7474076; doi:10.1038/s41408-020-00357-4)

## Supplementary Appendix (File description: Definitions, baseline characteristics, summary of deaths, and duration of response)

### Overall survival

Overall survival was defined as time from randomization to the date of death from any cause. Patients who did not die had their survival time censored at the date of last contact (last known alive date). Patients were censored at the date of randomization if they were randomized but had no follow-up.

### Progression-free survival (PFS)

The primary analysis of PFS was based on the primary definition per independent review committee (IRC) assessment. Supportive analyses for PFS were: IRC assessment and intent-to-treat (ITT) definition; PFS per investigator assessment and primary definition; and PFS per investigator assessment and ITT definition.

#### Primary definition of PFS

The time from randomization to the date of first documented tumor progression or death due to any cause. Patients were censored at the last adequate assessment prior to the start of any subsequent systemic therapy or at the last adequate assessment prior to 2 missing assessments (>10 weeks). Patients who died more than 10 weeks after the randomization date and had no on-treatment assessment were censored at the randomization date. Clinical deterioration was not considered progression.

The following censoring rules were applied for PFS: patients who received secondary anti-myeloma therapy prior to documented progression were censored on the date of the last tumor assessment prior to the initiation of the new therapy; patients who had an event (documented progression or death) >10 weeks (2 assessment visits) after the last prior tumor assessment were censored at the last prior assessment; patients who neither received subsequent therapy prior to progression nor had a progression event were censored at their last tumor assessment.

#### ITT definition of PFS

The time from randomization to the date of first documented tumor progression or death due to any cause. Patients who did not have any post-baseline tumor assessments and did not die were censored on the date of randomization.

### Supplemental Table 1 Demographics and baseline characteristics.

| **Characteristic** | **ERd (n = 321)** | **Rd (n = 325)** |
| --- | --- | --- |
| **Age, median (range), years** | 67 (37–88) | 66 (38–91) |
| **ISS stage at diagnosis** |  |  |
| I–II | 243 (76) | 243 (75) |
| III | 66 (21) | 68 (21) |
| **Cytogenetic profile** |  |  |
| del(17p) | n = 315**^a^** | n = 322**^a^** |
| ≥1 cell | 102 (32) | 104 (32) |
| t(4;14) | n = 315**^a^** | n = 321**^a^** |
| ≥1 cell | 30 (10) | 31 (10) |
| **Risk category** | n = 305**^a^** | n = 309**^a^** |
| High^b^ | 60 (20) | 66 (21) |
| Standard^c^ | 231 (76) | 221 (72) |
| Low^d^ | 14 (5) | 22 (7) |
| **Prior therapy regimens** |  |  |
| Median (range) | 2 (1–4) | 2 (1–4) |
| 1 | 151 (47) | 159 (49) |
| ≥2^e^ | 170 (53) | 166 (51) |
| **Previously received agents** |  |  |
| Bortezomib | 219 (68) | 231 (71) |
| Thalidomide | 153 (48) | 157 (48) |
| Lenalidomide | 16 (5) | 21 (6) |
| **Prior stem cell transplantation** | 167 (52) | 185 (57) |
| **Response to most recent line of therapy^f^** |  |  |
| Refractory | 113 (35) | 114 (35) |
| Relapsed | 207 (64) | 211 (65) |

Data are *n* (%) unless otherwise specified.

^a^Patients evaluable for cytogenetic abnormality/risk category; percentages were calculated based on evaluable patients.

^b^ISS stage II or III and t(4;14) or del(17p) abnormality.

^c^Patients not meeting the definition of high or low risk.

^d^ISS stage I or II and absence of t(4;14), del(17p), and 1q21 abnormalities and age <55 years.

^e^1 patient in each arm had received >3 prior regimens.

^f^Response for 1 patient in the ERd arm was unknown.

*ERd* elotuzumab plus lenalidomide and dexamethasone, *ISS* International Staging System, *Rd* lenalidomide and dexamethasone.

Adapted from *New England Journal of Medicine*, Lonial S et al. Elotuzumab therapy for relapsed or refractory multiple myeloma, 373, 621–631. Copyright © 2015 Massachusetts Medical Society. Reprinted with permission

### Supplemental Table 2 Summary of deaths.

| **Disposition** | **ERd (n = 318)** | **Rd (n = 317)** |
| --- | --- | --- |
| Total deaths | 212 (67) | 225 (71) |
| **Primary cause of death** |  |  |
| Disease progression | 131 (41) | 142 (45) |
| Infection | 28 (9) | 20 (6) |
| Cardiovascular disease | 12 (4) | 16 (5) |
| Other malignancy/neoplasm | 9 (3) | 6 (2) |
| Study drug toxicity | 7 (2) | 7 (2) |
| Bleeding | 2 (1) | 6 (2) |
| Other/unknown | 23 (7) | 28 (9) |

Data are *n* (%).

### Supplemental Fig. 1 Duration of response.

### *CI* confidence interval; *DoR* duration of response; *ERd* elotuzumab plus lenalidomide and dexamethasone; *Rd* lenalidomide and dexamethasone.


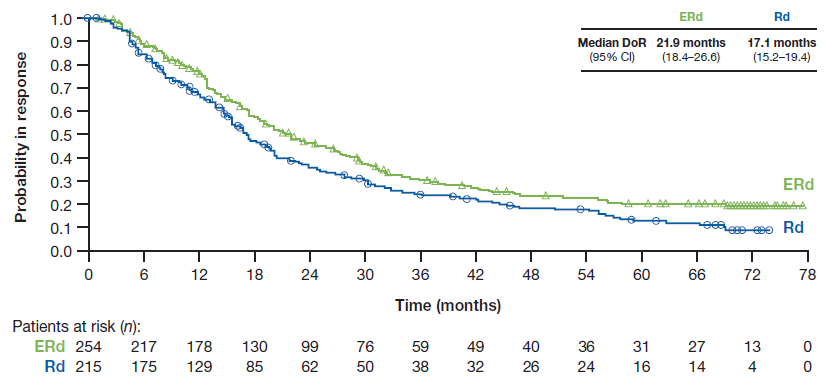

Supplement: Supplementary file 1 — Supplementary Appendix [file 41408_2020_357_MOESM1_ESM.docx]
